# Photodynamic Therapy as an Oxidative Anti-Tumor Modality: Negative Effects of Nitric Oxide on Treatment Efficacy

**DOI:** 10.3390/pharmaceutics13050593

**Published:** 2021-04-21

**Authors:** Albert W. Girotti, Jonathan M. Fahey, Mladen Korbelik

**Affiliations:** 1 Department of Biochemistry, Medical College of Wisconsin, Milwaukee, WI 53226, USA; 2 Department of Pathology, University of Colorado, Aurora, CO 80045, USA; jonfahey@gmail.com; 3 Department of Integrative Oncology, BC Cancer, Vancouver, BC V5Z 1L3, Canada

**Keywords:** photodynamic therapy, singlet oxygen, photooxidative stress nitric oxide, inducible nitric oxide synthase, S-nitrosation, tumor cell resistance, tumor cell aggressiveness, bystander effects, BET proteins, BET inhibitors

## Abstract

Anti-tumor photodynamic therapy (PDT) is a unique oxidative stress-based modality that has proven highly effective on a variety of solid malignancies. PDT is minimally invasive and generates cytotoxic oxidants such as singlet molecular oxygen (^1^O_2_). With high tumor site-specificity and limited off-target negative effects, PDT is increasingly seen as an attractive alternative or follow-up to radiotherapy or chemotherapy. Nitric oxide (NO) is a short-lived bioactive free radical molecule that is exploited by many malignant tumors to promote cell survival, proliferation, and metastatic expansion. Typically generated endogenously by inducible nitric oxide synthase (iNOS/NOS2), low level NO can also antagonize many therapeutic interventions, including PDT. In addition to elevating resistance, iNOS-derived NO can stimulate growth and migratory aggressiveness of tumor cells that survive a PDT challenge. Moreover, NO from PDT-targeted cells in any given population is known to promote such aggressiveness in non-targeted counterparts (bystanders). Each of these negative responses to PDT and their possible underlying mechanisms will be discussed in this chapter. Promising pharmacologic approaches for mitigating these NO-mediated responses will also be discussed.

## 1. Introduction: Photodynamic Therapy

**Figure 1:**
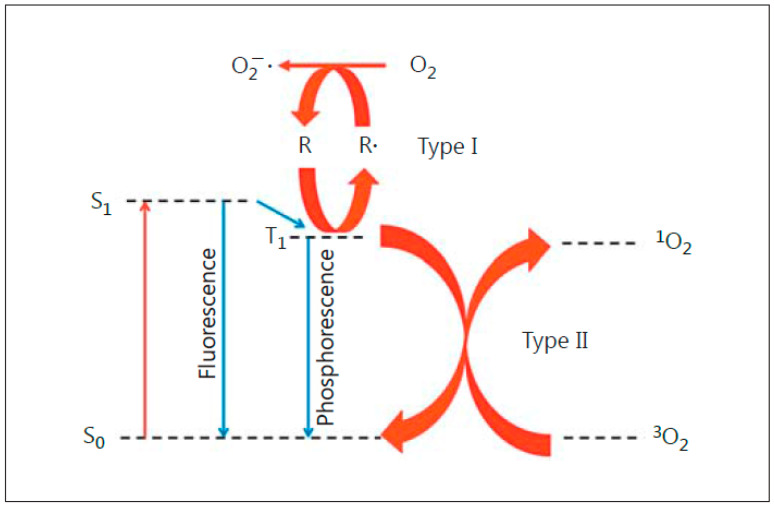
Photoexcitation of a sensitizing agent to a relatively long-lived triplet state (T_1_), which can initiate Type I (free radical-mediated) or Type II (singlet oxygen-mediated) reactions. Reproduced with permission from [4], Karger, 2015.

Anti-tumor photodynamic therapy (PDT) was introduced about 45 years ago as a novel clinical approach for selectively eradicating a variety of malignant solid tumors via cytotoxic photochemistry [1,2,3,4]. Many of these tumors were refractory to conventional chemotherapy or radiotherapy. PDT is a minimally invasive modality which exhibits little, if any, off-target cytotoxicity. Classical PDT consists of three operating components: (i) a photosensitizing agent (PS), (ii) PS photoexcitation by non-ionizing radiation (typically in the far visible-to-near infrared wavelength range), and (iii) molecular oxygen [2,3,4]. All three components must be engaged concurrently to activate the photodynamic process, and light delivery via fiber optic networks makes PDT highly selective for an intended tumor target. In 1995, Photofrin^®^, a hematoporphyrin oligomer, became the first PS to receive Federal Drug Administration approval for clinical PDT, with esophageal malignancies being treated initially [2]. Since then, PDT with Photofrin^®^ and other PSs has been used on numerous other malignancies, including prostate, breast, cervical, head-and-neck, and brain (gliomas) [3,4]. Most PSs are innocuous until photoactivated and, unlike many chemotherapeutic agents, have few (if any) negative effects on normal tissues. A common photodynamic reaction in PDT (Type II process) involves energy transfer from photoexcited triplet state PS to ground state O_2_ (Figure 1), giving singlet molecular oxygen (^1^O_2_), a cytotoxic short-lived reactive oxygen species (ROS). For some PSs, electron transfer to O_2_ may occur (Type I process), resulting in the formation of superoxide (O_2_^−^•) and other free radical ROS (Figure 1). Similarly to ^1^O_2_, the latter can kill tumor cells by oxidizing vital proteins and lipids and/or activating death signaling pathways [3,4,5]. In addition to preexisting PSs, which are administered directly in various carriers, pro-PSs have been developed which can be converted to active PSs in target tumors. One prominent example is 5-aminolevulinic acid (ALA), which is metabolized to protoporphyrin IX (PpIX) via the heme biosynthetic pathway [6,7]. To accommodate pro-growth/expansion activity, this pathway is typically more active in transformed cells than normal counterparts, thus accounting (at least in part) for greater PpIX accumulation in the former [6,7]. PpIX builds up initially in mitochondria and, upon photoactivation, induces photodamage therein, often leading to cell death via intrinsic apoptosis [8]. PDT with well-established PSs such as Photofrin^®^, benzoporphin derivative (BPD), silicon phthalocyanine (Pc4), and ALA-induced PpIX usually hits cytoplasmic targets (lysosomes, endoplasmic reticulum, mitochondria) [3,4]. In contrast, radiotherapy and chemotherapy usually affect nuclear targets, such as cisplatin, causing DNA cross-linking [9,10]. Since PDT acts mainly in the cytoplasm, this may explain its effectiveness as a follow-up or combined treatment with DNA-targeted chemo- or radiotherapy. It is well known that many tumors exhibit an intrinsic or acquired resistance to the latter modalities, and this is now evident for PDT as well. For example, a PDT challenge may upregulate antioxidant or anti-apoptotic proteins [11]. Moreover, some cancer cells can upregulate membrane transporter ABCG2, thereby reducing PDT efficacy by exporting ALA-induced PpIX and other PSs [12]. PDT is also known to be compromised by endogenous nitric oxide (NO) produced by inducible nitric oxide synthase (iNOS). In this chapter, we will discuss (i) the ability of iNOS-derived NO at low steady state levels to antagonize PDT, (ii) the underlying mechanisms of iNOS/NO upregulation by PDT stress, (iii) the possible mechanisms of NO-elicited resistance to PDT, (iv) the role of NO in hyper-aggressiveness of bystander cells; and (v) how these negative responses can be mitigated by pharmacological interventions that suppress NOS/NO.

## 2. Nitric Oxide and Its Role in Cancer

NO is a short-lived bioactive free radical molecule with a half-life of <2 s in H_2_O [13]. It diffuses freely on its own in aqueous media and, like O_2_, tends to partition into low polarity membrane zones in cells [14,15,16]. Naturally occurring NO is generated by three enzymes in the nitric oxide synthase (NOS) family: neuronal (nNOS) and endothelial (eNOS), which both require available Ca^2+^ for optimal activity, and inducible (iNOS), which does not require Ca^2+^ [17,18]. All NOS isoforms catalyze the five-electron oxidation of L-arginine to L-citrulline and NO at the expense of NADPH and O_2_ [17,18]. At steady state levels in the millimolar range, NO produced by activated macrophages can be toxic to established tumor cells, but may also be mutagenic and carcinogenic [14,15,16,19,20]. However, in the low-to-medium nanomolar range, NO can play a key role in tumor persistence and progression by activating oncogenic signaling pathways or inhibiting tumor suppression pathways [19]. For malignant tumors, NO in the lower range can promote growth and metastatic expansion as well as resistance to therapeutic agents [19,20]. Cancer cell iNOS/NO can activate signaling cascades mediated by proteins such as soluble guanylyl cyclase (sGC), hypoxia-inducible factor-1α (HIF-1α), epidermal growth factor receptor (EGFR), phosphoinositide-3-kinase/protein kinase B (PI3K/Akt), and extracellular signal-regulated kinases-1/2 (ERK1/2) [15,19,20,21,22]. There is evidence for a gradation in the signaling effectiveness of low-level NO. For example, 1–10 nM NO stimulates cell proliferation via sGC activation, whereas 0.3–0.4 μM NO does so via activation of the EGFR/PI3K/Akt pathway [21,23]. iNOS/NO and tumor suppressor p53 have been shown to act in opposing fashion in many tumors. For example, wild type p53 can block iNOS transcription or bind and deactivate the enzyme [24]. Conversely, NO has been reported to chemically modify p53, thereby inhibiting its pro-apoptotic activity [25]. It is also known that tumor cells with dysfunctional (mutated) p53 express higher levels of iNOS than wild-type controls, and this correlates with more aggressive growth and migration in the former [26]. Based on extensive evidence, therefore, the iNOS level in tumor samples is now considered a reliable prognostic indicator of cancer survival, with patients with the highest levels given the worst chances and vice versa [9,20]. How might one explain NO’s pro-tumor activity in mechanistic biochemical terms? One possible mechanism involves S-nitrosation of specialized cysteine residues on key effector proteins. The relatively low pK_a_ value of these cysteine -SH groups increases their likelihood of S-nitroso (SNO) modification [27,28,29]. Consistent with a transient signaling role, SNO groups can be removed by glutathione or thioredoxin as oxidative pressure subsides [29]. Effector proteins susceptible to SNO modification include (i) the pro-apoptotic MAP kinases JNK and ASK1, which are inhibited [30]; (ii) antiapoptotic Bcl-2, whose proteasomal degradation is inhibited [31]; (iii) phosphatase MKP-1, which is protected against degradation, allowing pro-apoptotic JNK and p38 to be inactivated [32]; and (iv) tumor suppressor PTEN, which is inactivated [33]. Each of these SNO modifications might support tumor persistence and progression.

## 3. Role of Endogenous NO in Tumor Resistance to PDT

How endogenous NO might affect the anti-tumor potency of PDT at the in vivo level was first investigated about 20 years ago by two different groups using mouse syngeneic tumor models and Photofrin^®^ as PS. Henderson et al. [34] found that PDT for mouse-borne RIF tumors was much improved when L-NNA, a competitive inhibitor of NOS activity, was administered before and after irradiation. At about the same time, Korbelik et al. [35,36] showed that the PDT cure rate for RIF and SCCVII tumors, but not EMT6 or FsaR tumors, could be significantly improved when L-NNA or L-NAME (another non-specific NOS inhibitor) was introduced immediately after irradiation. Importantly, RIF and SCCVII tumors were found to produce NO at a much higher constitutive rate than EMT6 or FsaR tumors, thus accounting for the greater inhibitor effects on the first two [36]. RIF and SCCVII tumors with relatively high NO outputs exhibited lower intrinsic sensitivity to PDT than the others, suggesting that basal NO measurements might serve as useful predictors of PDT efficacy [36]. More recently, Reeves et al. [37], also using mouse syngeneic tumors, but ALA-induced PpIX as PS, confirmed that tumor-generated NO exerted a negative effect on anti-tumor PDT. Essentially the same deduction was made in each of these studies, viz. that vasodilation due to endothelium-derived NO (presumably eNOS NO) was counteracting PDT’s well-known tumor-abating vasoconstrictive effects, allowing better maintenance of tumor oxygen supply during treatment. Although the earliest iteration of this work [34,35,36] was ground-breaking with regard to PDT antagonism by NO in vivo, it left the following questions unsettled: (i) whether the NO derives from tumor cells alone or whether surrounding cells (endothelial, macrophages, fibroblasts) or tumor-invading, PDT-induced cells like neutrophils might contribute; (ii) which NOS isoform is most important in any given tumor; (iii) whether the NOS/NO in question functions at a pre-existing level or is upregulated by PDT stress; and (iv) the biochemical mechanisms that underlie NO’s anti-PDT effects. Results of in vivo and in vitro studies dealing with these issues will be discussed in the following sections.

## 4. Possible Mechanisms of NO-Mediated Resistance: In Vivo Studies

NO and derived reactive nitrogen species can either cause reversible S-nitrosation (also called S-nitrosylation, incorrectly) of protein thiol groups (see Section 2) or irreversible tyrosine nitration in targeted proteins [38]. In the former case, NO in the nanomolar range can act as a cellular signaling molecule by reversible redox-based modification of cysteine residues on targeted proteins and in this way, it regulates a multitude of physiological and pathophysiolgical processes. Depending on NO levels under cellular stress conditions, S-nitrosation may either be conducive to a cell death program or serve in a negative feedback mechanism that inhibits pro-death effects [39].

**Figure 2:**
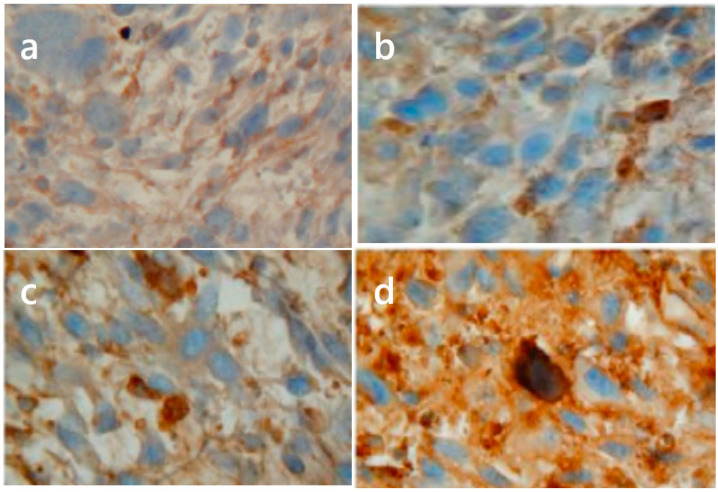
Immunohistochemical detection of S-nitrosated proteins in tumors after temoporfin-PDT. Mouse FsaR fibrosarcomas growing in syngeneic C3H/HeN mice were PDT treated (temoporfin 0.1 mg/kg iv. followed 24 h later by 35 J/cm^2^ of 650 ± 10 nm light) as described previously [42]. Tumor tissue sections (5 μm) collected at 1 (**b**), 2 (**c**), and 4 (**d**) hours after PDT light treatment, as well as control (untreated) tumors (**a**) were stained with rabbit antiserum to S-nitrocysteine (Alexis Biochemicals), following manufacturer’s instructions. Magnification: 400×. (M. Korbelik, unpublished data).

The bioavailability of NO and its interaction with cell macromolecules may depend on its rapid reaction with superoxide radical (O_2_^−^•) to produce peroxynitrite (ONOO^−^), a potent mediator of protein oxidation and nitration, as well as lipid peroxidation [5,14]. Superoxide, generated by the one-electron reduction of molecular oxygen, is one of the ROS that may be formed during PDT [3]. Reaction of O_2_^−^• with NO gives ONOO^−^ and thence nitronium cation (NO_2_^+^) and NO_2_ radical, comprising a major pathway of protein tyrosine nitration. However, another pathway exists, which depends on myeloperoxidase (typically from activated neutrophils) as it converts NO-derived nitrate to nitryl chloride and nitrogen dioxide [40]. Tyrosine nitration, which can be detected by anti-nitrotyrosine antibodies, serves as a “footprint” of the reactive nitrogen species generated in cells. This technique has been used to demonstrate the induction of nitrosative stress in sarcoma-bearing rats following PDT treatment or local hyperthermia [41]. On the other hand, Korbelik et al. showed that S-nitrosation also occurs in PDT-treated tumors. This was demonstrated in FsaR fibrosarcomas growing in syngeneic mice that were subjected to temoporfin-sensitized PDT and recovered for analysis at various post-irradiation times (Figure 2). The treated tumors were subjected to immunohistochemical analysis based on staining with antiserum to S-nitrocysteine.

As shown in Figure 2, SNO-cysteine staining increased over time and was particularly strong at 4 h after the PDT challenge. At present, however, the identity of the SNO-modified proteins is unknown. It remains unresolved whether S-nitrosation has an overall positive or negative effect on PDT-mediated tumor cure rates. On the one hand, S-nitrosation has been reported to have pro-tumor effects, while on the other hand, elevating it has been found to inhibit tumor growth and stimulate cell death [29].

It is well established that PDT treatment induces a strong and rapid invasion of neutrophils in treated tumors [41]. Sluiter et al. [43] suggested that the elevated iNOS activity detected in PDT-treated tumors [43] can at least in part be due to invading activated neutrophils. Consequently, the rise in NO levels in these tumors can to some extent be attributed to these neutrophils. The increase in tumor blood flow known to be induced after PDT may also be dependent on NO released from invading neutrophils [36]. This PDT-induced increase in tumor blood flow was shown to be largely subdued in neutrophil-depleted mice [44].

**Figure 3:**
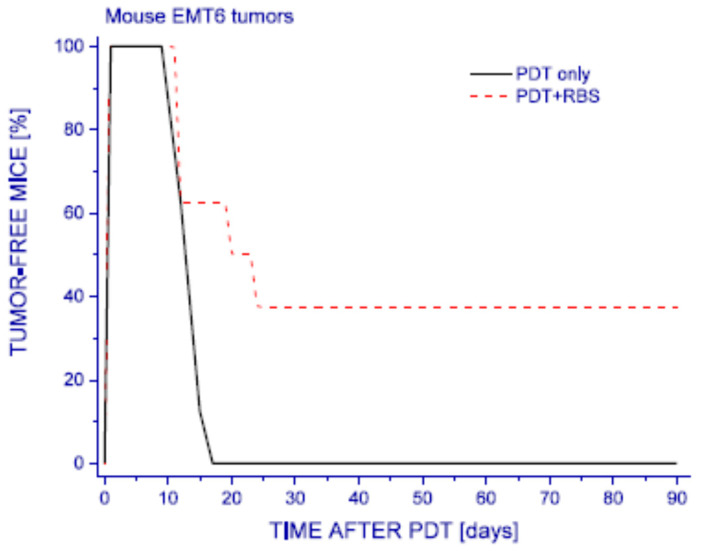
Roussin’s black salt (RBS)-enhanced efficacy of tumor PDT. Mouse EMT6 mammary sarcomas were treated with Photofrin-PDT (Photofrin: 5 mg/kg; 120 J/cm^2^ of 630 ± 10 nm light; fluence rate: 100 mW/cm^2^). One group of tumor-bearing mice was also given RBS (20 μmol/kg, *ip.*) 30 min before PDT light. This light-induced, NO releasing drug was provided by Drs. J. Bourassa and P. Ford (Dept. of Chemistry, University of California-Santa Barbara). The mice were observed afterwards for signs of tumor recurrence and those remaining tumor-free at 90 days post PDT were considered cured. The treatment with RBS alone provided no significant effect on tumor response (not shown). The difference in response between PDT-only and PDT + RBS group is statistically significant (*p* < 0.05). (M. Korbelik, unpublished data).

The notion that rapid site-specific delivery of NO in massive doses can lead to necrotic cell death can be exploited for increasing the efficacy of anti-tumor PDT. This approach could be achieved by combining PDT with light-activated NO donors such as Roussin’s black salt (RBS) [45], as shown in Figure 3. In this case, PDT irradiation served an additional function by inducing the tumor-localized release of NO in high fluxes from the donor. This combined treatment clearly resulted in a substantially greater tumor cell kill than PDT alone. Thus, while PDT alone produced no permanent cures, combining it with the NO donor increased the cure rate to ~40%. Hence, the advantage of this type of approach is clear.

## 5. Role of iNOS/NO in Tumor Resistance to PDT: In Vitro and In Vivo Studies

**Figure 4:**
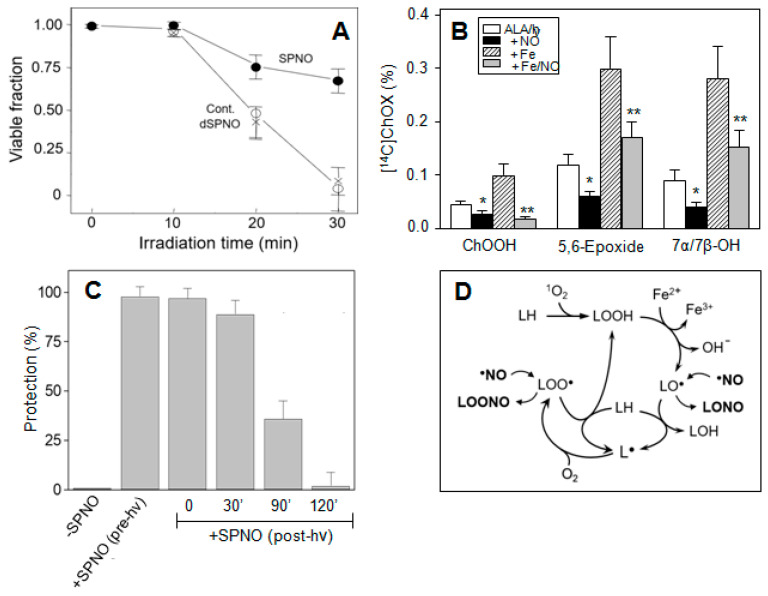
Nitric oxide-imposed resistance to free radical-mediated photokilling of human breast cancer cells. COH-BR1 cells in DME/F12 medium were membrane-labeled with [^14^C]cholesterol, then dark-incubated with 1 mM ALA for 15 min, followed by 215 min without ALA. During the latter, most of the ALA-induced PpIX diffused from mitochondria to plasma membrane. After switching to fresh medium lacking or containing SPNO (0.4 mM), cells were irradiated for the indicated times (light fluence rate: 1.2 mW/cm^2^). (**A**) Ho258-assessed viability at 5 h post-irradiation. ALA/light (○), ALA/SPNO/light (•), ALA/spent SPNO/light (x); mean values (*n* = 2). (**B**) Various [^14^C]ChOX species determined for cells irradiated as in (**A**), but lacking or containing Fe(HQ)_3_ (0.5 μM). ChOOH: total cholesterol hydroperoxide. Plotted values: means ± deviation (*n* = 2); * *p* < 0.05 vs. ALA/hν; ** *p* < 0.05 vs. ALA/Fe/hν. (**C**) Post-irradiation effects of NO on cell viability. SPNO (0.4 mM) was either absent or added immediately after irradiation or at indicated post-hν times. Plot: protection relative to viability of dark controls. (**D**) Scheme showing ^1^O_2_-mediated formation of primary LOOHs, chain peroxidation induced by iron-catalyzed LOOH reduction, and NO interception of lipid-derived radicals. Reproduced with permission from [54], Wiley, 2003.

Most PSs of PDT-relevance, including ALA-induced PpIX, are amphiphilic and tend to localize at membrane-aqueous interfaces. This property facilitates their ability to sensitize peroxidation of unsaturated membrane phospholipids, glycolipids, and cholesterol [5]. Photoactivation of PpIX, for example, typically gives rise to ^1^O_2_, which can react directly with unsaturated lipids to give lipid hydroperoxides (LOOHs). If reductants (e.g., glutathione, ascorbate) and redox-active Fe(III) are available, these primary LOOHs are likely to undergo one-electron reduction to highly reactive free radical intermediates (LO∙/OLOO∙). These intermediates will induce chain peroxidation of surrounding lipids, thereby expanding and exacerbating the membrane damaging effects of primary LOOH formation [5]. Lipophilic antioxidants such as α-tocopherol and β-carotene protect against such damage by scavenging chain-promoting, lipid-derived radicals [46]. Studies by Rubbo et al. [47,48] using xanthine oxidase- or lipoxygenase-catalyzed peroxidation of liposomal or lipoprotein lipids revealed that NO can also act as a highly efficient inhibitor of free radical-mediated lipid peroxidation. This was demonstrated for NO delivered in gaseous form or released from chemical donors. A chain-breaking antioxidant mechanism was demonstrated whereby NO rapidly intercepts LOO∙/LO∙ intermediates to give non-radical LOONO/LONO species. Somewhat later, Kelley et al. [49] found that exogenous NO could also protect leukemia HL-60 cells against cytotoxic lipid peroxidation induced by ferrous iron. These findings [47,48,49] prompted Girotti and co-investigators to determine whether NO could act similarly in the context of PDT. Initial studies involved unilamellar liposomes composed of unsaturated phospholipids, cholesterol, and a primary or “priming” LOOH. The selected LOOH was cholesterol 5α-hydroperoxide (5α-OOH), a definitive ^1^O_2_ adduct previously isolated from photoperoxidized liposomes [50]. Upon incubation with a lipophilic iron chelate, ferric-8-hydroxyquinoline [Fe(HQ)_3_] and a reductant, ascorbate (AH), the 5α-OOH decayed in first-order fashion and in the process triggered damaging chain peroxidation mediated by LO∙/OLOO∙and LOO∙ [50,51]. When the NO donor, spermine-nonoate (SPNO), was present from the outset, the rate of chain peroxidation, as reported by free-radical-induced cholesterol 7α/7β-hydroperoxide (7α/7β-OOH), was dramatically reduced [51]. Fully decomposed SPNO had no effect, implying that NO from active SPNO was functioning as a chain-breaking antioxidant. It is important to point out that cholesterol-derived hydroperoxides (ChOOHs) such as 5α-OOH and 7α/7β-OOH were detected and quantified by means of high-performance liquid chromatography with mercury cathode electrochemical detection (HPLC-EC(Hg)), an ultra-high sensitivity/specificity analytical approach developed in the Girotti laboratory [52]. In addition to ChOOHs, HPLC-EC(Hg) has been used to detect and determine phospholipid counterparts (PLOOHs) isolated from photooxidized membrane systems [52]. The extent of chain peroxidation and how it is affected by NO can also be assessed by high-performance thin layer chromatography with phosphor-imaging detection (HPTLC-PI), another high-sensitivity/specificity approach developed by Girotti and co-investigators [53]. In HPTLC-PI, radiolabeled cholesterol ([^14^C]Ch) is incorporated into a membrane of interest and serves as a probe for free radical-mediated reactions occurring in its midst [53]. After extracted lipids are subjected to TLC, separated [^14^C]ChOOHs and other cholesterol oxides ([^14^C]ChOX species) are quantified by PI. This approach was first used with liposomal models and subsequently with human COH-BR1 cells, a human breast cancer subline [54,55]. The cells were metabolically sensitized with PpIX by first incubating with ALA in serum-free medium, then allowing most of the porphyrin to diffuse from mitochondria to plasma membrane, turning it into a highly sensitive primary target [54,55]. Irradiation with broad-band visible light resulted in a progressive loss of viability which reached ~50% after 5 h in the dark following 20 min. of light exposure (Figure 4A). The cells died by necrosis, consistent with membrane-breaching lipid peroxidation. When active, but not decomposed SPNO was introduced immediately before irradiation, photokilling was markedly diminished, suggesting that NO protected the cells by inhibiting chain peroxidation. Additional supporting evidence was obtained by labeling COH-BR1 plasma membranes with [^14^C]Ch and measuring [^14^C]ChOX levels. As shown in Figure 4B, ALA/light-induced increases in [^14^C]ChOX (total ChOOH and 5,6-epoxide and 7α/7β-OH end-products) were significantly reduced by SPNO-derived NO. This was also the case when chain peroxidation was stimulated by a trace amount of Fe(HQ)_3_, and it confirmed that in a PDT model system, NO had acted as a chain-breaking antioxidant. The results shown in Figure 4C reveal that significant amounts of cytoprotection could still be observed if SPNO was added at different points after irradiation instead of immediately before, as done in the experiment shown in Figure 4A. For example, protection was ~90% when SPNO entered the system at 30 min post-irradiation, and ~35% when it entered 60 min later. Thus, NO inhibition of lethal peroxidative damage did not cease after a given irradiation period (10 min in this case), but continued long thereafter, although to a diminishing extent. These results demonstrate the striking “momentum” of post-irradiation chain peroxidation. Surprisingly, many PDT practitioners are still unaware of this light-independent aftermath. The scheme in Figure 4D depicts ^1^O_2_-mediated LOOH formation, chain peroxidation induced by iron-catalyzed LOOH reduction, and NO action as a chain breaking antioxidant.

**Figure 5:**
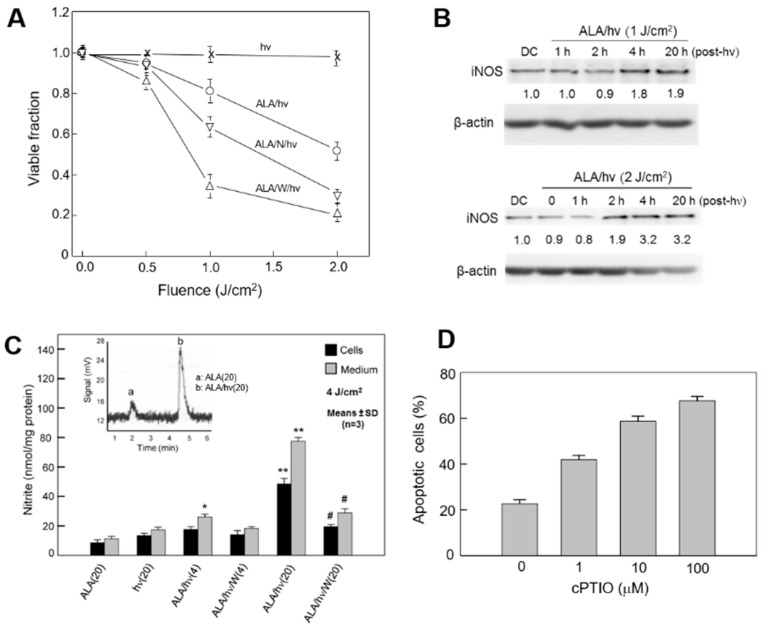
Mitochondria-targeted photokilling of breast cancer cells: protective effects of endogenous iNOS/NO. COH-BR1 cells in serum-free medium were sensitized with PpIX in mitochondria by dark-incubating with 1 mM ALA for 45 min, then switched to ALA-free medium without or with L-NAME (1 mM) or 1400W (10 μM), and exposed to increasing light fluences up to 2 J/cm^2^. (**A**) MTT-assessed viability 20 h after switching irradiated cells to 1% serum-containing medium; means ± SD (*n* = 3) (**B**) iNOS Western blots for surviving cells at 1, 2, 4, and 20 h after the indicated light fluences; DC, dark control. (**C**) Chemiluminescence-based determination of NO in cells and cell media 4 and 20 h after ALA/light compared with ALA-only or light-only control. * *p* < 0.005 vs. ALA(20).** *p* < 0.0001 vs. ALA(20). ^#^ *p* < 0.001 vs. ALA/hv(20). (**D**) cPTIO-enhanced apoptotic photokilling; means ± SD (*n* = 3). Reproduced from [54], Wiley, 2003. Reproduced with permission from [55], Elsevier; 2010.

The above evidence in this section was based on an exogenous source of NO, i.e., outside the photodynamically challenged cancer cells. Since many tumor cells generate NO to support survival and growth/migratory expansion, a crucial question is how this endogenous NO might affect PDT, e.g., ALA-based PDT. In initial experiments, breast COH-BR1 cells sensitized in mitochondria with ALA-induced PpIX were exposed to increasing light in the absence vs. presence of L-NAME or 1400W (an iNOS-specific inhibitor), and then examined for viability after 24 h of dark incubation. As shown in Figure 5A, the extent of photokilling was increased by L-NAME and more so by 1400W, suggesting that iNOS/NO was imposing significant resistance.

Immunoblot analyses of surviving cells (still attached) indicted that they not only expressed iNOS, but upregulated it progressively during post-irradiation incubation. The extent of upregulation was light fluence-dependent, reaching ~2 and ~3 times the control level after 20 h for 1 and 2 J/cm^2^, respectively (Figure 5B). The magnitude of upregulation for each light dose was found to be statistically significant for several replicate experiments [55]. Chemiluminescence-based NO analysis confirmed that iNOS-derived NO was elevated in ALA/light-treated cells. As shown in Figure 5C, a 1400W-inhibitable NO signal was observed in both cells and cell medium. This signal intensified during post-irradiation incubation up to 20 h, consistent with the observed upswing in iNOS level (Figure 5B). Mitochondrial PpIX-sensitized COH-BR1 cells died mainly via intrinsic apoptosis after irradiation. As shown in Figure 5D, the extent of apoptosis increased progressively when a NO scavenger, cPTIO, was present in increasing concentrations during irradiation. Clearly, therefore, photodynamic stress-induced NO was signaling for resistance to apoptotic cell death. Several other human cancer lines, including prostate PC3, glioblastoma U87, and triple negative breast MDA-MB-231 cells have been found to exhibit similar NO-mediated resistance to ALA/light-induced photokilling [56,57,58,59]. In each case, this was accompanied by a rapid and prolonged upregulation of iNOS protein, e.g., ~8-fold for still attached PC3 cells at 20 h after irradiation [57].

These in vitro findings were recently extended to the in vivo level, using female immunodeficient (SCID) mice engrafted with MDA-MB-231 tumors [59]. After intraperitoneal ALA administration, animal tumors were subjected to irradiation using a 633 nm LED source. Tumor size was significantly reduced after PDT treatment compared with non-ALA (light-only) controls. However, when an iNOS inhibitor (1400W or GW274150) was administered before and after irradiation, tumor size decreased even further [59], suggesting that iNOS/NO was imposing resistance to PDT, as had been observed for MDA-MB-321 cells in vitro. iNOS inhibitors had no effect on control tumors, suggesting that pre-existing iNOS/NO was not essential for tumor persistence. Immunoblotting of post-PDT tumor samples revealed a striking upregulation of iNOS which, after 6 h, reached ~5-fold above the unchanged light-control level. Analysis for NO-derived nitrite revealed a strong 1400W-inhibitable increase in this parameter after PDT [59]. This was the first known evidence for increased resistance associated with iNOS/NO induction in a human tumor PDT model.

## 6. Role of iNOS/NO in Hyper-Aggressiveness of PDT-Surviving Tumor Cells

**Figure 6:**
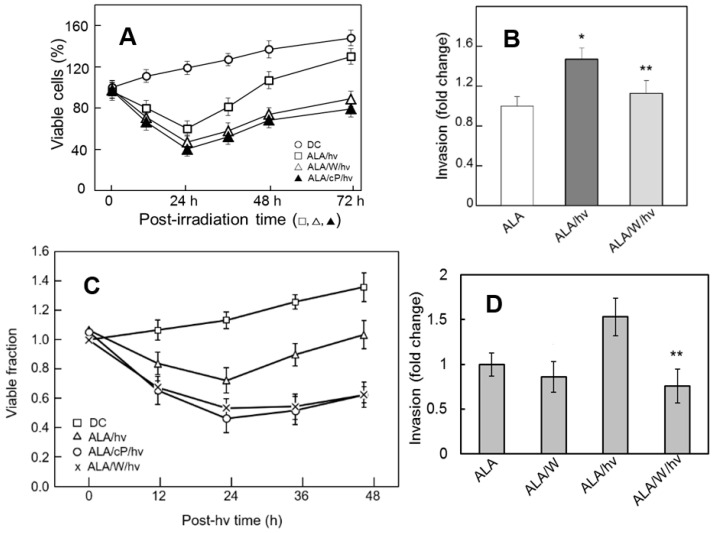
Post-ALA/light survival, proliferation, and invasion properties of prostate and brain cancer cells: iNOS/NO-dependent enhancements. (**A**) Prostate PC3 cells were subjected to an ALA/light challenge (1 J/cm^2^) in the absence vs. presence of 25 μM 1400W (W) or 25 μM cPTIO (cP); DC: ALA-only-dark control. At varying post-irradiation times up to 72 h, viable cell count relative to time zero was determined by MTT assay; means ± SD (*n* = 3). (**B**) Surviving PC3 invasiveness over a 48 h post-irradiation period: effects of 1400W; * *p* < 0.05 vs. ALA; ** *p* < 0.05 vs. ALA/hν. (**C**) Glioblastoma U87 cells were exposed to ALA/light (1 J/cm^2^) in the absence vs. presence of 1400W or cPTIO as in (**A**), after which viability was measured over 48 h. (**D**) Surviving U87 cell invasiveness; means ± SD (*n* = 3); ** *p* < 0.01 vs. ALA/hν. Reproduced with permission from [57], Elsevier, 2015. Reproduced from [58], Wiley, 2016.

Due to various complexities, including non-uniform PS or pro-PS distribution and non-uniform delivery of PS-exciting light, not all cancer cells in any given tumor will be lethally damaged by PDT. This situation can be mimicked in vitro by using relatively modest PS and/or light doses such that a significant number of cells remain attached and viable after a PDT-like challenge. Fahey and Girotti [57,58,59] discovered that these cells typically exhibit more aggressive behavior than non-challenged controls. This hyper-aggressiveness has been demonstrated for several human cancer cell types and is manifested by more rapid proliferation, migration, and invasion, each of which is fostered by upregulated NOS/NO. When PC3 cells were monitored over a 3-day period after ALA/light exposure, the viable count decreased progressively, reaching ~60% after 24 h (Figure 6A). When present, 1400W or cPTIO (a NO scavenger) caused a more rapid loss in viable count, which approached ~40% at 24 h. iNOS was upregulated ~8-fold over this time [57], explaining the NO-based resistance revealed by 1400W and cPTIO. This resistance was against apoptotic photokilling, as had been observed for COH-BR1 and MDA-MB-231 cells [59]. When surviving PC3 cells were monitored beyond the 24 h point, a striking (~3-fold) increase in proliferation rate was observed relative to dark (ALA-only) controls (Figure 6A). This growth spurt persisted from at least 24 to 48 h of post-irradiation incubation, but was abolished by 1400W or CPTIO, signifying iNOS/NO involvement (Figure 6A). Flow cytometric cell cycle analysis indicated increasingly greater (but 1400W-inhibitable) S-phase occupancy over a 36 h post-irradiation period. It is clear from these and related findings [57] that DNA doubling time in surviving cells was promoted by iNOS/NO, consistent with their greater proliferation rate. Of added importance was the discovery that photostress-surviving PC3 cells exhibited a remarkable increase in motility as manifested by migration and invasion. As shown in Figure 6B, invasiveness of ALA/light-treated cells, as assessed by trans-membrane (Boyden-type) assay, was at least 50% greater than that of ALA-only controls, and 1400W strongly inhibited this response, implicating iNOS-derived NO as the major driving agent [57]. Similar evidence has been obtained for prostate carcinoma DU-145 cells [57]. Enhanced aggressiveness in response to photodynamic stress has also been observed for other cancer types, e.g., glioblastoma U87 cells. After a mitochondria-centered ALA/light challenge, U87 cells underwent an increasing loss of viability via apoptosis during subsequent dark incubation up to 24 h (Figure 6C). As observed with PC3 cells, U87 apoptosis was also stimulated by 1400W or cPTIO, consistent with iNOS/NO-imposed resistance. Further incubation of surviving (still attached) cells beyond the 24 h point revealed a growth spurt lasting at least another 24 h, the rate being about twice that of control cells (Figure 6C). This spurt was effectively abolished by 1400W or cPTIO, demonstrating major iNOS/NO dependency, as was deduced for the PC3 growth spurt. A large increase in U87 invasiveness was also observed, which was strongly blunted by 1400W (Figure 6D). However, 1400W had no significant effect on control invasiveness, suggesting that the strong increase in photostressed cells was due to iNOS-generated NO, the enzyme being upregulated ~3-fold in these cells [58]. Although significant nNOS was also expressed, it was not upregulated by ALA/light treatment [58], suggesting that stress-induced iNOS was unique in supplying NO for stimulation of growth and migratory aggressiveness.

Matrix metalloproteinases (MMPs) such as MMP-9 catalyze the degradation of collagen and other extracellular matrix (ECM) components, and thus play a key role in cancer cell invasiveness and metastasis [60]. Inherent migration and invasion of many tumor cells is known to be stimulated by MMP-9, which becomes activated by proteolytic cleavage of its externalized precursor, pro-MMP-9 [60,61]. Fahey et al. [57,58] found that MMP-9 activity for ALA/light-stressed PC3 and U87 cells was consistently much higher than that of dark controls, and that iNOS inhibitors blocked this increase. Of added importance was the observation that a tissue inhibitor of metalloproteinases (TIMP-1) was progressively down-regulated in photostresssed PC3 cells, and in a 1400W-inhibitable fashion [57], thus revealing an intricate iNOS/NO-controlled relationship between MMP-9 and TIMP-1 which favored more aggressive invasiveness. Several other proteins that play important roles in tumor expansion exhibited iNOS/NO-dependent upregulation in photostressed PC3 and U87 cells: integrins α6 and β1, survivin, and S100A4 [57,58]. Collectively, these findings demonstrate the cooperative action of several effector proteins in a broad network that fosters tumor cell migration/invasion. It is important to point out that the studies described [57,58] focused exclusively on natural intracellular iNOS/NO as opposed to chemical donor NO or transfected iNOS used in many other studies dealing with NO’s tumor supporting/expanding properties. Thus, the evidence presented in this section is more realistic regarding iNOS/NO involvement, particularly in the context of anti-tumor PDT.

## 7. Role of NO in PDT-Induced Bystander Effects

As indicated above, most established tumors have defective vascular systems. Therefore, not all tumor cells would be uniformly supplied with an administered PS or pro-PS such as ALA. Moreover, during subsequent irradiation, some cells might only be exposed minimally or not at all due to insufficient light coverage, limited penetration, and scattering. Nevertheless, one could imagine that cells experiencing the greatest photodynamic stress might send signals to non- or minimally stressed neighboring cells (bystanders). Bystander effects are well documented for cancer cells exposed to ionizing radiation [62,63,64], and at least two means of signal transmission have been described: (i) via gap junctions between cells, and (ii) via the medium without actual cell contact [64]. Evidence for the latter has become more available as analytical methods have improved. Various signaling molecules capable of traversing aqueous media have been identified for X- or γ-irradiation, including cytokines [65], H_2_O_2_ [66], and NO [65,67,68,69]. Although NO has a short half-life and must be continuously generated to be effective, it differs from H_2_O_2_ in having no known enzymatic scavengers. PDT-induced bystander effects were first described about 20 years ago [70] and followed up 10 years later [71,72]. In the latter work, several signaling intermediates other than NO were proposed, e.g., H_2_O_2_, and LOOHs. The first studies to consider NO as a mediator of PDT bystander effects were carried out by Bazak et al. [73]. A novel silicone ring-based approach was used for initially separating ALA/light-targeted PC3 cells from non-targeted PC3 bystanders [73]. The rings were removed at some point after irradiation (typically 2–3 h), allowing any stress-upregulated factors to move from targeted cells to naïve bystanders. There was no physical contact between the two populations, so diffusion of signaling molecules through the medium was the only means of intercellular communication. As anticipated from earlier findings [56,57], photostress-withstanding target cells overexpressed iNOS, and its NO stimulated their proliferation and migration. Strikingly similar responses were observed in non-stressed bystanders, i.e., 1400W- and cPTIO-inhibitable iNOS/NO upregulation and faster proliferation and migration. Since the conditioned medium from targeted cells did not elicit these bystander responses [73], it was concluded that NO played the dominant role. Several other tumor-supporting proteins besides iNOS were upregulated in PC3 bystanders, including cyclooxygenase-2 (COX-2) and protein kinases Akt and ERK1/2. Once again, targeted cell iNOS/NO was primarily responsible [73]. These seminal studies on PC3 cells have recently been extended to three other human cancer lines: melanoma BLM, breast MDA-MB-231, and brain U87 [74]. Pre-incubation with ALA was the same for all these cells, but light fluences were varied, meaning that a uniform target cell kill was attained, i.e., ~25% at 24 h after irradiation. iNOS in both cell compartments underwent progressive upregulation over 24 h after irradiation, but to the following extents: PC3 > MDA-MB-231 > U87 > BLM. Proliferation and migration rates of targeted and bystander cells were enhanced in the same order. For example, BLM cells with the lowest iNOS induction exhibited the smallest increase in proliferation/migration rate, whereas PC3 cells with the greatest induction exhibited the greatest rate increase [74]. Thus, bystander aggressiveness increased in direct proportion to the extent of iNOS and NO upregulation in targeted cells that survived the ALA/light insult. A type of relay process is envisaged from these [74] and earlier findings [73], whereby NO overproduced in targeted cells diffuses to naïve bystanders and induces iNOS/NO there. How incoming NO does this in the bystander compartment, is still unknown. These findings suggest a NO “feed-forward” phenomenon that propagates through the bystander population. Such possibilities raise concerns about the tumor-promoting potential of bystander effects if they occur during clinical PDT.

## 8. Mechanism of iNOS Upregulation by Photodynamic Stress

Bhowmick and Girotti [75], using breast COH-BR1 cells, and Fahey et al. [76], using glioblastoma U87 cells, found that transcription factor NF-κB plays a key role in ALA/light-induced iNOS upregulation. Consistent with this, there was a rapid transfer of NF-κB subunit p65/RelA from cytosol to nucleus of photostressed cells for initiation of iNOS transcription [75,76]. Based on non-PDT studies of others [77], it was postulated that acetylation of specific lysine residue(s) on p65 is required for stimulating it as a transcriptional co-activator. Supporting evidence revealed that lysine-310 on p65 was increasingly acetylated (p65-acK310) in ALA/light-challenged U87 cells [76]. Upon interrogation of upstream signaling events, it was found that p65-acK310 formation was dependent on activation of pro-tumor kinases PI3K and Akt, followed by phosphorylation-activation of acetyltransferase p300. Supporting these sequential activations was the inactivation of tumor-suppressor PTEN, a PI3K antagonist, via intramolecular disulfide bond formation [76]. Elevation of acK310 level by ALA/light treatment was strongly suppressed by C646, an inhibitor of activated p300, confirming the latter’s role in K310 acetylation. Of added importance was the photostress-induced downregulation of sirtuin-1, a deacetylase that regulates gene expression by removing acetyl groups from histones and transcription factors [78]. Along with these responses, there was a striking upregulation of type-4 bromodomain protein (Brd4), an epigenetic reader and transcriptional co-activator for iNOS and other stress-responsive genes [76]. Moreover, K310 acetylation promoted the interaction of Brd4 with p65 for transcriptional stimulation [76]. These findings reveal a remarkably well-coordinated and cooperative upstream signaling network set in motion by photodynamic stress and leading ultimately to overexpression iNOS/NO for enhancement of tumor cell survival and progression. The involvement of key stress signaling cascades and unfolded protein response in the control of NF-κB expression is also well evidenced [79].

## 9. Pharmacologic Approaches for Mitigating NO’s Anti-PDT Effects

As indicated above, identification of iNOS/NO signaling for a survival, proliferative, and migratory/invasive advantage in tumor cells is typically based on the suppressing effects of specific inhibitors of iNOS activity (1400W, GW274150) or NO scavengers (e.g., cPTIO). Would these pharmacologic agents be similarly effective at the clinical level if used in conjunction with anti-tumor PDT? This appears not to have been attempted yet, but there is good reason to believe that certain iNOS inhibitors would improve clinical PDT efficacy. Two such inhibitors, L-NIL and GW274150, have already been used in clinical trials [80,81], but these had no relationship to cancer or PDT. Instead, the inhibitors were tested for relieving inflammation associated with asthma and neither one had any negative side effects. As indicated in Section 5, GW274150 significantly improved PDT efficacy in breast tumor xenograft model [59], suggesting that this inhibitor might be a good candidate as a PDT adjuvant. Inhibitors of bromodomain/extra-terminal domain (BET) proteins such as Brd4 were introduced about 10 years ago as powerful new means of suppressing tumor persistence and progression at the transcriptional level [82]. These inhibitors function by binding to BET domains, thereby preventing interaction with acK groups on transcription factors (e.g., NF-κB-p65-acK310) or on histones [82,83]. When one such inhibitor, JQ1, at sub-toxic concentration (~0.3 μM), was tested on ALA/light-stressed U87 cells, it (i) synergized with photostress in killing these cells, (ii) strongly inhibited Brd4 interaction with p65-acK310, (iii) prevented iNOS upregulation by photostress, and (iv) prevented surviving cells from becoming hyper-aggressive [84]. Importantly, JQ1 inhibited these negative effects at a concentration ~100-fold lower than that of 1400W or GW274150, making JQ1 much more promising as a PDT adjuvant, especially since it has already been used successfully in conjunction with other anti-cancer therapies [85,86]. Transcriptional upregulation of pro-tumor iNOS/NO in conjunction with PDT and other anti-tumor therapies based on oxidative stress may occur more often than presently realized, thus emphasizing the need for highly effective BET inhibitors like JQ1 as treatment adjuvants.

## 10. Summary and Perspectives

We have discussed the basic principles of PDT and the advantages it has over other anti-tumor therapies based on oxidative stress. As with other modalities, PDT is often confronted with pre-existing or stress-induced resistance, which inevitably reduces treatment efficacy. As highlighted in this review, low-flux NO generated by tumor cell iNOS plays a major role, not only in this acquired resistance, but also in the enhanced proliferative and migratory aggressiveness of cells that can withstand the photooxidative challenge. We have also described how NO from PDT-targeted cells can induce iNOS/NO in non-targeted bystander cells, making them more aggressive via a NO “feed-forward” process. For most of the in vitro and in vivo studies described, this challenge was imposed by photoexcitation of ALA-induced photosensitizer PpIX in mitochondria. From substantial evidence with different types of human cancer cells, it is now clear that the anti-PDT effects described are not significantly dependent on NO from pre-existing iNOS, but rather NO generated by photostress-upregulated enzyme. Levels of the latter typically remained highly elevated for several hours after photodynamic treatment. These findings are quite unique because for most tumor therapies up to now, the possibility of iNOS overexpression during treatment has not been considered, and nor has the possibility that any NO from upregulated iNOS might be more antagonistic than that from the pre-existing enzyme. We described the underlying mechanism of iNOS induction by photostress for one cancer cell type (glioblastoma), which presumably holds for other types as well. Although less is known about how the resulting NO imposes greater resistance or aggressiveness, some mechanistic information is available. Regarding hyper-resistance, we described NO’s ability to inhibit membrane-damaging lipid peroxidation by intercepting lipid-derived radicals. On the other hand, NO might promote survival, growth, and migration/invasion via signaling pathways. S-nitrosation of specialized cysteine residues on pro-survival vs. anti-survival effector proteins falls into this category, and we provided data from in vivo PDT samples showing that S-nitrosation does occur. Clearly, however, much more needs to be learned about the mechanisms by which iNOS-derived NO can antagonize PDT. Concerns about more aggressive and possibly more metastatic phenotypes of PDT-surviving cells could be mitigated by using inhibitors of iNOS enzymatic activity or iNOS transcription, as pointed out. In the latter category, we discussed the advantages of BET inhibitors like JQ1, and anticipate that administering them as clinical PDT adjuvants will greatly improve treatment efficacy.

## Data Availability

All pertinent previously unpublished data is contained within the article (Figure 2 and Figure 3).

## Abbreviations

PDT, photodynamic therapy; NO, nitric oxide; iNOS, inducible nitric oxide synthase; PS, photosensitizer; ALA, 5-aminolevulinic acid; PpIX, protoporphyrin IX; ROS, reactive oxygen species; ^1^O_2_, singlet molecular oxygen; O_2_^−^•, superoxide anion radical; 1400W, N-[3 (aminomethyl)benzyl]acetamidine; cPTIO, 2-(4-carboxyphenyl)4,4,5,5-tetramethylimidazoline-1-oxyl-3-oxide; SNO, S-nitroso; BET, bromodomain and extra-terminal domain; JQ1, (*S*)-*tert*-butyl 2-(4-(4-chlorophenyl)-2,3,9-trimethyl-6*H*-thieno[3,2-*f*][1,2,4]triazolo[4,3-*a*][1,4]diazepin-6-yl) acetate.
